# Optimal Sequential Immunization Can Focus Antibody Responses against Diversity Loss and Distraction

**DOI:** 10.1371/journal.pcbi.1005336

**Published:** 2017-01-30

**Authors:** Shenshen Wang

**Affiliations:** Department of Physics and Astronomy, University of California Los Angeles, Los Angeles, California, United States of America; Harvard University, UNITED STATES

## Abstract

Affinity maturation is a Darwinian process in which B lymphocytes evolve potent antibodies to encountered antigens and generate immune memory. Highly mutable complex pathogens present an immense antigenic diversity that continues to challenge natural immunity and vaccine design. Induction of broadly neutralizing antibodies (bnAbs) against this diversity by vaccination likely requires multiple exposures to distinct but related antigen variants, and yet how affinity maturation advances under such complex stimulation remains poorly understood. To fill the gap, we present an in silico model of affinity maturation to examine two realistic new aspects pertinent to vaccine development: loss in B cell diversity across successive immunization periods against different variants, and the presence of distracting epitopes that entropically disfavor the evolution of bnAbs. We find these new factors, which introduce additional selection pressures and constraints, significantly influence antibody breadth development, in a way that depends crucially on the temporal pattern of immunization (or selection forces). Curiously, a less diverse B cell seed may even favor the expansion and dominance of cross-reactive clones, but only when conflicting selection forces are presented in series rather than in a mixture. Moreover, the level of frustration due to evolutionary conflict dictates the degree of distraction. We further describe how antigenic histories select evolutionary paths of B cell lineages and determine the predominant mode of antibody responses. Sequential immunization with mutationally distant variants is shown to robustly induce bnAbs that focus on conserved elements of the target epitope, by thwarting strain-specific and distracted lineages. An optimal range of antigen dose underlies a fine balance between efficient adaptation and persistent reaction. These findings provide mechanistic guides to aid in design of vaccine strategies against fast mutating pathogens.

## Author Summary

Highly mutable pathogens pose significant challenges to vaccine design, mainly owing to the vast antigenic diversity they present to the immune system. Recently an increasing variety of broad antibodies that can recognize diverse strains have been isolated from patients, but how to induce them by vaccination is largely unknown. In particular, how affinity maturation, the Darwinian process that evolves potent antibodies, proceeds under multiple stimulations by distinct antigen variants is not well understood. We use computer simulations and evolutionary models to examine realistic new aspects important for vaccine development: loss of B cell diversity in between immunization periods and the existence of distracting molecular features that do not contain conserved elements. We find counterintuitive impact of these factors on antibody breadth development, which depends crucially on temporal arrangements of selection forces. Our findings provide guides for optimal vaccination strategies and reveal their evolutionary basis.

## Introduction

**Fig 1:**
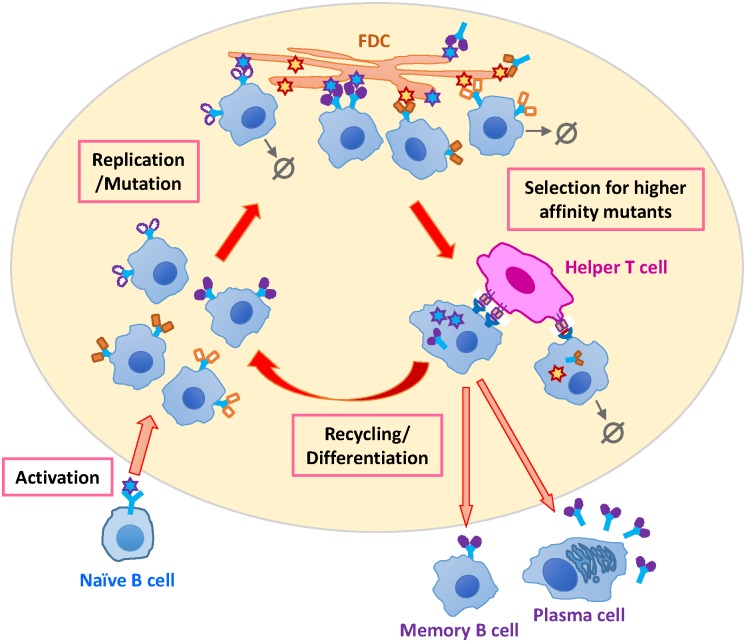
Schematic depiction of B cell affinity maturation. Upon activation, naïve B cells seed a germinal center. They mutate their antigen-receptor-encoding genes as they replicate. Mutated B cells that bind sufficiently strongly to their encountered antigen on the FDCs can internalize it and present derived short peptides to helper T cells. Higher affinity B cells can engulf and process more antigen and present a greater amount of peptides and thus compete better for T cell help. Selected B cells mostly recycle to mature further, and the rest differentiate and exit the germinal center as memory cells or antibody-secreting plasma cells. B cells that fail to get activated or receive T cell help are lost via apoptosis (zero symbols).

Upon infection or vaccination, antibodies (Abs) are generated through affinity maturation (AM), a Darwinian process occurring in a short time (Fig 1). Affinity maturation mainly takes place in germinal centers (GCs), which are dynamic structures in secondary lymphoid tissues that arise and dissolve in response to antigen (Ag) stimulation [1, 2]. GCs house B cells and T helper cells, as well as resident f ollicular dendritic cells (FDCs) that present antigens to B cells. Somatic hypermutation diversifies the Ag receptors of B cells as they replicate. Mutated B cells that bind Ag sufficiently strongly can internalize it and present short peptides derived from pathogenic proteins bound to major histocompatibility complex (MHC) class II molecules on their surface. T helper cells can potentially bind to these peptide-MHC molecules to provide a survival signal. While Ag displayed on FDCs is the fuel that sustains GC reactions (GCRs), limited T cell help drives competition between B cells. Through rounds of mutation and selection, GC B cells can enhance the Ag affinity of their receptors up to 10^3^ folds within a few weeks [3]. Most selected B cells are recycled for further rounds of mutation and selection [4, 5]. The rest differentiate into memory and plasma cells. Soluble forms of the B cell receptors (BCRs) secreted by plasma cells are called Abs.

Highly mutable complex pathogens, such as HIV, have evolved mechanisms to evade immune recognition as well as divert immune responses, such that they can persist in a circulating population and diversify. Therefore, a protective Ab response must cover a very diverse pool of viral strains. Recently, an increasing number and variety of broadly neutralizing antibodies (bnAbs) have been isolated from chronically infected patients [6–10]. These bnAbs can individually recognize a vast majority of global viral isolates. Notably, potent monoclonal bnAbs have dramatic effects on blocking viral transmission [11, 12] and controlling (though transiently) established infection [13, 14] in non-human primates. These findings have renewed the hope for an effective HIV vaccine because they provide proof that the human immune system can evolve such Abs. But attempts to elicit HIV bnAbs efficiently by vaccination have so far been unfruitful [6, 8, 9, 15].

Recent longitudinal tracking of HIV-1 bnAb lineages and the co-evolving virus, in infected individuals over more than a few years has suggested that a broadening reactivity of elicited Abs relies on an expanding diversity of the encountered Ag [16–20]. This observation proves that AM can indeed lead to broad Abs, but also emphasizes a tardy development of bnAbs in a non-protective amount by natural immunity. Importantly, it points to the necessity of using multiple Ag variants as immunogens. This finding has triggered a shift in focus from AM against a single Ag, extensively studied by experiment [3, 5, 21–30] and models [4, 31–37], to investigating how AM occurs when multiple distinct Ag variants are present [38–41]. The latter situation is poorly understood.

Vaccination with multiple variants of a complex Ag provides an opportunity (and also a daunting combinatorial complexity) to design the selection forces acting on B cell populations during AM, and to arrange them in an optimal temporal sequence. Our recent work [39] on vaccine strategies against HIV has shown that sequential immunization with Ag variants (that share a set of conserved residues, but whose variable residues are separated by relatively large mutational distances) is favored over a simultaneously administered cocktail of the same variants in inducing bnAbs. This is in line with previous immunization studies [42, 43] and was tested in model experiments in mice [39]. New experiment [44] provides further support for the sequential strategy. The key lies in temporal separation of potentially conflicting selection forces represented by the variant Ags which can frustrate affinity maturation.

Apart from its remarkable variability, HIV has complex molecular features that present unique challenges to vaccination. The protective effect of an Ab is determined by how well it binds to a set of residues, the epitope, on the proteins that make up the spike on the surface of the virion. The HIV-1 envelope glycoprotein trimer (Env) is the sole target of known HIV-1 neutralizing Abs. These trimeric spikes are made of proteins that are among the most mutable in the viral proteome. But they do contain highly conserved residues that are essential for mediating viral entry by binding to particular receptors expressed on host cells (e.g. HIV has to bind to CD4 receptors to infect helper T cells). However, these conserved residues are often surrounded by highly variable regions and masked by glycans and variable protein loops that hamper access to the conserved residues [45–49]. Recent modeling studies involving multiple Ags [38, 41, 50] have assumed independent epitopes, either purely conserved or entirely variable, which occupy separate locations on the viral protein. Computational [41] and analytical [50] studies on co-evolution of Abs and HIV viruses using such a model have indicated that increasing the *initial* diversity of Ag variants and the antigenic distances between them makes it more likely that a bnAb lineage establishes. We believe that this result, at least in part, emerges from the assumption that epitopes are either entirely conserved or entirely variable. It is crucial to consider concurrent conserved and variable residues in the target epitope as this reflects reality and poses an additional challenge to bnAb induction.

Despite the apparent similarity in successive presentations of distinct Ag variants, there are important differences between the dynamics of natural infection and vaccination. Infection by mutating viruses presents a continuously varying selective pressure, which sustains the GC reaction as new viral mutants establish before their ancestors get cleared. In contrast, upon vaccination by distinct Ag variants well separated in time, GCs are likely to dissolve prior to the arrival of a new variant. Reseeding of GCs then raises a new question: to what extent is B cell passed across successive periods of immunization, and how this affects bnAb evolution.

As has recently been demonstrated, properly designed immunogens can induce germline B cells that target epitopes containing the conserved residues on the HIV viral spike proteins [51–53]. But, it seems likely that to evolve bnAbs one would have to subsequently immunize with variant Ags that resemble the viral spike (such as variants of SOSIP [47, 48]). In addition to the target epitope containing the conserved residues, such immunogens (and the Env spikes) also present numerous distracting epitopes that are easily accessible, highly immunogenic and yet do not contain conserved residues [54]. Abs directed towards these epitopes are not able to recognize even close variants of them and thus will have very narrow reactivity. How such distraction impacts the efficacy of bnAb induction in various vaccination schemes is important, and yet unexplored.

Aiming to design vaccination strategies to favor HIV bnAb evolution, we extend our previously developed model of AM driven by variant Ags to explicitly account for the process of GC reseeding upon immunization with new variant Ags, and the existence of distracting epitopes. This stochastic dynamic model enables a detailed investigation of the evolutionary mechanisms behind divergent maturation outcomes in various immunization schemes. We describe how different temporal arrangements of selection forces shape evolutionary dynamics of competing B cell lineages that in turn determine the predominant mode of Ab responses that emerge. We find that sequential immunization with mutationally distant yet related Ag variants can induce bnAbs, and is also most robust to distraction. Unexpectedly, diversity loss in between periods of immunization may even favor bnAb evolution. Our results also highlight the prominent role of Ag dose in balancing the seemingly incompatible demands of adaptation efficiency and persistence of maturation. Therefore, this study provides mechanistic guides that may aid the design of vaccination strategies that can induce bnAbs against highly mutable complex pathogens.

## Model

### Affinity maturation in a germinal center

We employ a coarse-grained stochastic model of B cell AM in the GC (Fig 1). Naïve B cells acquire genetically diverse receptors via recombination events (VDJ recombination), which create a diversity in their ability to bind different epitopes. Upon activation, *N*_sd_ = 3 distinct naïve clones seed each GC and replicate without mutation to ∼1500 cells [24]. This mimics the growth stage before the enzyme responsible for hypermutation of the BCR variable region genes turns on. Cyclic action of mutation and selection ensues, driving the evolution of B cells. Point mutations are introduced uniformly at a rate of 0.5 per sequence per division [21]. The probability that a mutation is functionally silent (no change in affinity) is *p*_*S*_ = 0.5, the probability of a lethal mutation (e.g. non-folding) is *p*_*L*_ = 0.3, and the rest are affinity-affecting mutations (probability *p*_*A*_ = 0.2) [32]. Affinity change due to point mutations is drawn from an asymmetric bounded distribution, with deleterious mutations that reduce affinity more likely [37, 39]. We model the dynamics of B cell populations as branching processes, which naturally accommodate time-varying GC sizes. Population bottleneck (BN) arises because overwhelming detrimental mutations first lead to population decline, and only after rare beneficial mutations emerge and establish, population growth starts, giving rise to even more favorable mutants that populate a GC. We assume all the maturing B cells in a GC are synchronized in replicating, making mutations, competing for survival and recycling or output. GC reaction ends either when all B cells die, when a threshold number of B cells survive (i.e., ∼1500 cells), or when a maximum duration is reached (120 days or 240 GCR cycles), whichever comes first. The last two termination conditions reflect Ag uptake by the maturing GCs or Ag decay, respectively. As a consequence, mature GCs may differ in population size and duration of reaction. Persistent maturation at intermediate population sizes allows B cells to sample and accumulate the large number of mutations required for high affinity and large breadth.

B cell survival requires success in two stochastic processes. Mutated B cells can internalize Ag if their BCR binds sufficiently strongly to it [27]. The probability of this event is modeled as obeying Langmuir equilibrium at a physiological temperature T:

$$P_{a}^{i}=\frac{\sum_{j=1}^{n_{A}}C_{j}e^{(E_{j}^{i}-E_{a})/kT}}{1+\sum_{j=1}^{n_{A}}C_{j}e^{(E_{j}^{i}-E_{a})/kT}},$$ (1)

where $E_{j}^{i}$ is the affinity of B cell *i* to Ag of type *j* and *E*_*a*_ denotes the activation threshold. *C*_*j*_ is the concentration of Ag of type *j* presented on the FDCs. The sum over the Ag index *j* runs through *n*_*A*_ distinct types that B cell *i* can potentially interact with simultaneously during an encounter with a FDC bearing multiple Ag variants. Activated B cells internalize the Ag and present derived short peptides bound to MHC molecules on their surface to attract attention from helper T cells. B cells with higher affinity for Ag obtain and process a greater amount of Ag in a given period of time and thus outcompete the surrounding B cells by presenting more peptide-MHCs to helper T cells. There has been increasing evidence showing that competition between B cells for limited T cell help is essential for establishment and persistence of GC reactions [5, 29]. It is clear that probabilities of being activated (*P*_a_) and of receiving T cell help (*P*_Th_) are both dictated by the binding affinity of a B cell to its encountered Ag. Thus, we model the probability of a B cell to succeed in receiving T cell help by

$$P_{\text{Th}}^{i}=\frac{W_{i}}{W_{i}+R{\left\langle W_{i^{\prime }}\right\rangle }_{i^{\prime }\left(\neq i\right)}}.$$ (2)

Here $W_{i}=\sum_{j=1}^{n_{A}}C_{j}e^{E_{j}^{i}/kT}$ is in proportion to the probability of B cell *i* internalizing Ag, which is compared to the average probability, 〈*W*_*i*′_〉_*i*′(≠*i*)_, over all the competing B cells. The parameter *R* is a dimensionless quantity of order 1, which is related to the total Ag concentration and the ratio between B cells and T helper cells. Since the precise dependence is unknown, we compute it as $R={\left(\sum_{j=1}^{n_{A}}C_{j}\right)}^{-1}$, which accounts for the observation that a higher Ag dose triggers a stronger response of T helper cells, thus making it more likely that B cells receive T cell help.

The level of spatial heterogeneity of Ag display on FDCs remains unclear. We consider two scenarios of Ag presentation: (1) Ag variants are homogeneously distributed on the FDCs, and thus B cells interact simultaneously with all types of variants in each contact with the FDCs, namely “See all Ag”; (2) Ag distribution is heterogeneous and each B cell interacts with only one type of Ag variant randomly chosen in each round of selection, which we call “See 1 Ag”.

### Ag variants with complex epitopes

**Fig 2:**
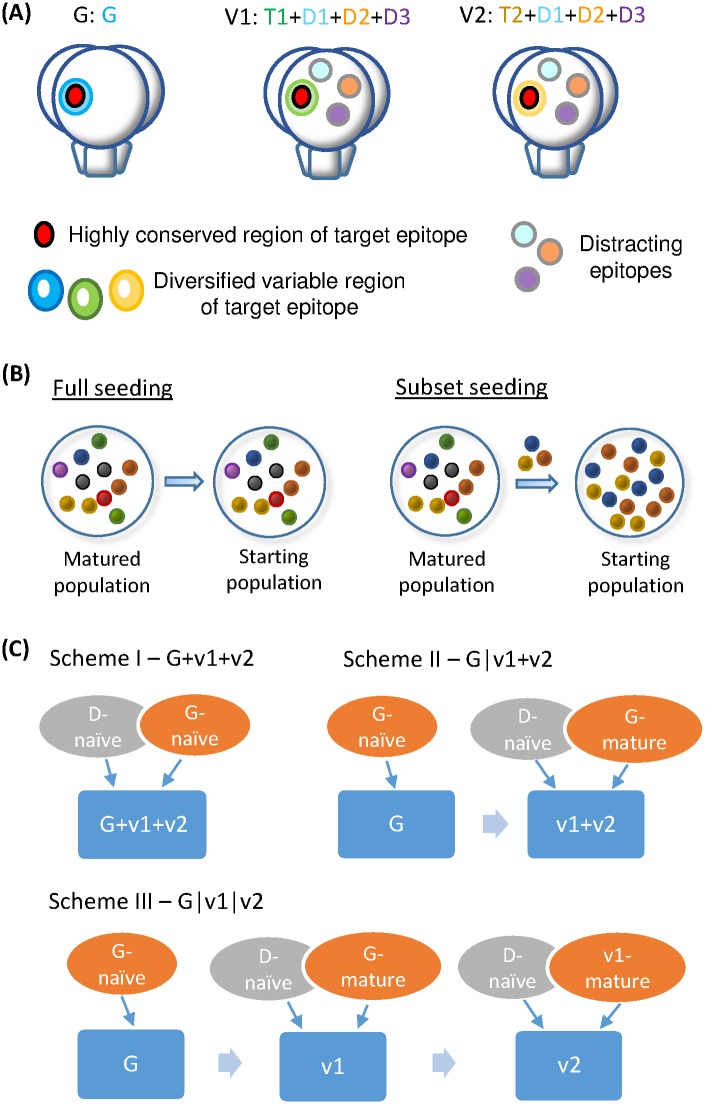
Model antigen variants and seeding schemes. (A) Cartoon of three antigen variants in the trimeric form of the HIV Env protein (side view). The variant G has only G epitope, whereas variants v1 and v2 have distinct target epitopes T1 and T2, respectively, as well as shared distracting epitopes D1, D2, and D3 (colored spherical regions). Target epitopes G, T1 and T2 have identical conserved part (red) and disparate variable parts (blue, green and yellow rings) which consist of a maximum number of non-overlapping mutations between them. The same set of epitopes is present on each monomer of a trimer. (B) Seeding schemes for a new GC reaction stimulated by a newly arriving antigen variant. For “full seeding” (left), the matured population is passed on as is, whereas for “subset seeding” (right), a handful of founder B cells are randomly drawn from the matured population and expanded in proportion to constitute a new GC population. Spheres in different colors represent distinct B cell clones. (C) Composition of seeding clones in various vaccination schemes. Naïve cells reactive to D epitopes (D-naïve) can join a nascent GC upon the advent of v1 and/or v2.

As a minimal set of immunogens to mimic the vast genetic diversity of HIV-1, we consider 3 Ag variants, G, v1 and v2, distinguished by the set of epitopes they carry (schematic in Fig 2A). Each variant has a unique target epitope (T epitope: G, T1 or T2), characterized by a maximum number of non-overlapping mutations between the variants (rings of different colors in Fig 2A). Importantly, target epitopes also contain an identical conserved part where mutations are not tolerable (the red oval area). The presence of a conserved core, surrounded by variable elements and glycans that limit access to the conserved residues, is a defining feature of the HIV epitopes that are targeted by broad and potent Abs [20, 55–57]. Note the variant G represents a reference strain which activates the desirable germline B cells [51–53] that recognize the G epitope, i.e., the autologous version of the target epitope.

To account for the molecular features of HIV-1 Env that do not contain conserved elements, we introduce 3 distracting epitopes (D epitopes, D1, D2 and D3) shared between the variants v1 and v2 (colored spherical regions in Fig 2A). Recognition of D eptiopes confers no breadth. D epitopes are constructed to be sufficiently distant from T epitopes in the sequence space that a B cell is unlikely to become cross-reactive to both. The number 3 is chosen to represent the greater abundance of D over T epitopes but is otherwise arbitrary. We assume the same set of D epitopes to be shared between v1 and v2 to present a most favorable situation for D-targeting clones, in which they have a constant supply of antigen and experience no frustration as v1 and v2 alternate. Therefore, this corresponds to a most distracting scenario for T-targeting clones. Either introducing distinct D epitopes to different Ag variants or raising the quantity of D epitopes would potentially frustrate D-targeting clones and reduce their distracting effect.

Now that each Ag variant carries multiple epitopes, target or distracting, the affinity of a B cell to its encountered Ag is determined by the epitope it binds most strongly to, among the available ones. This particular epitope is named the selecting epitope of the B cell. A B cell is noted as T-targeting if its selecting epitope is one of the target epitopes T1, T2 or G, otherwise D-targeting, by having maximum affinity to one of the distracting epitopes D1, D2 or D3. We assume individual B cells can always find their selecting epitopes, by performing diffusive search on the Ag surface to locate the best complementarity.

Each epitope residue is treated in a coarse-grained manner, such that it is either the wild-type (WT) amino acid or a mutant. The strength of interaction of a residue (*k*) on BCR (*i*) with a residue on the selecting epitope is denoted by $h_{k}^{i}$. The binding affinity, $E_{j}^{i}$, between a B cell clone ${\vec{h}}^{i}$ and an Ag variant ${\vec{s}}^{j}$ is modeled as

$$E_{j}^{i}\left({\vec{h}}^{i},{\vec{s}}^{j}\right)=\sum_{k=1}^{M}h_{k}^{i}s_{k}^{j}+\sum_{k=M+1}^{N}h_{k}^{i}.$$ (3)

The first *M* interactions are with variable residues on the selecting epitope, where *s*_*k*_ can be either 1 (WT) or −1 (mutated). The other (*N* − *M*) sites are conserved residues on the epitope with *s*_*k*_ = 1. The interaction strength *h*_*k*_ is drawn from a continuous and uniform distribution within a bounded range. For target epitopes, we assume an equal amount of conserved and variable residues (i.e., *M* = *N*/2), whereas for distracting epitopes, there are no conserved residues (i.e., *M* = *N*). As before [39], through pairwise correlated changes in *h*_*k* ≤ *M*_ and *h*_*k* > *M*_, we account for the fact that BCRs that reduce interactions with shielding or blocking variable residues are more likely to be able to access and make contacts with the protected conserved residues on the target epitope, as indicated in recent structural studies of bnAb-lineage members in complex with HIV-1 Env [20, 57]. Such non-local coupling effect pertinent to the three-dimensional structure of Abs provides a potential mechanism for maturing B cells to amplify their gain in affinity attained by point mutations and make large steps in affinity landscapes.

### Loss in diversity between periods of immunization and chance of distraction

Germinal centers are transient microstructures that disassemble once the supply of FDC Ag is exhausted, either by immune clearance or via self degradation. Therefore, upon successive exposures to distinct Ag variants well apart in time, new GCs arise in response to each new variant. This suggests that founder B cells of each nascent GC should be sampled from the memory pool formed against earlier variants.

Upon GC re-seeding, diversity bottleneck [25, 26] occurs prior to the onset of hypermutation. Since the extent of diversity loss is hard to probe experimentally and may vary from GC to GC, we consider the limiting cases of “full seeding” versus “subset seeding” (Fig 2B) which should encompass the biological conditions. “Full seeding” implies that the memory B cell pool from the previous round of maturation against variant Ags is passed on to the next maturation period in its entirety. Whereas for “subset seeding”, a modest number *N*_*sd*_ of seeding clones are either generated anew (i.e. naïve B cells reactive to D epitopes) or sampled from the most recent memory and evenly expanded to sum up to the initial GC size; here dramatic loss in diversity and marked changes in clonal composition may occur, since rare clones could be amplified by chance in newly formed GCs. Naïvely we might expect that diminishing diversity would slow adaptation, however as we will see, quite surprisingly, reducing the dominance of ancestral clones could allow a broader search for more advantageous mutants and even favor the evolution of bnAbs by sustaining the GC reaction.

We consider 3 immunization schemes as before [39]: Scheme I—a cocktail of three variants in one dose, G+v1+v2 (see 1 Ag or see all Ag), Scheme II—the germline-targeting variant followed by a mixture of two mutants, G|v1+v2 (see 1 Ag or see both Ag), and Scheme III—sequential administration of three variants, G|v1|v2. However, the composition of seeding clones now differs; since B cells reactive to D epitopes on v1 and v2 could also seed or join a GC when a new variant arrives (Fig 2C), Ab responses might be distracted from the target epitopes. For the *N*_*sd*_ seeding clones, we sample a random fraction from the most recent memory repertoire, and generate naïve cells for the rest that bind any of the D epitopes with above-threshold affinity. The degree of distraction in matured GCs turns out to vary significantly between schemes.

## Results

For each protocol, we simulate a large ensemble of GCs (∼10^3^ surviving GCs in each scheme under each condition). When all the GC reactions finish, we examine the affinity of each resulting Ab in a GC against each strain in the Seaman neutralization test panel [58, 59]. The breadth of coverage is defined as the fraction of test panel sequences that an Ab binds with an above-threshold affinity (which is slightly higher than the activation threshold). We use this definition of breadth, based on an actual panel of major HIV strains, to enable direct comparison with our previous findings [39] on relative efficacy of different immunization schemes, and therefore reveal the counterintuitive effect of new factors. We collect statistics from surviving GCs and present our results as histograms. These distributions can be viewed as the probability of inducing Abs with certain breadth in a typical individual.

### Loss in seeding diversity can sustain maturation and even facilitate adaptation

Sampling a handful of seeding clones from a diverse mature pool could dramatically alter the clonal composition of a GC population, from that shaped by evolution against previous Ag variant(s). By chance, B cells with rare binding patterns, lying in the tail of the affinity distribution in individual GCs, might get amplified through this initial partial sampling. Thus on the ensemble level, subset seeding could lead to stronger GC-to-GC variations compared to full seeding, as well as weaker dominance of ancestral clones. To focus on the effect of diversity loss alone, we examine these expectations and their implications in the absence of distracting epitopes.

**Fig 3:**
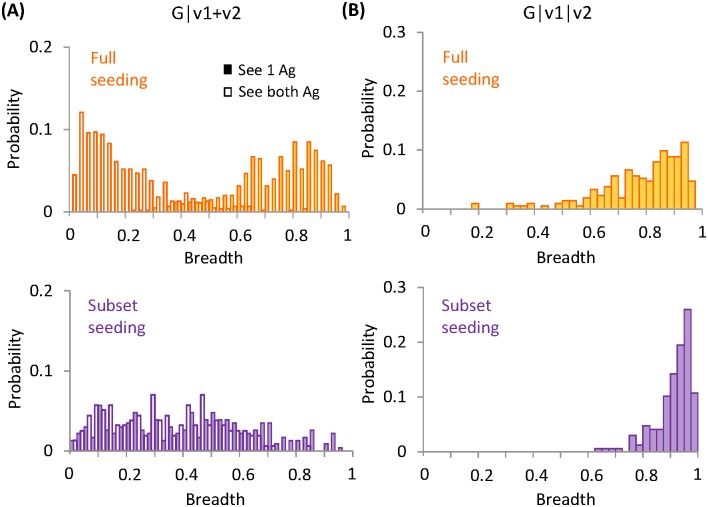
Effect of seeding diversity on antibody breadth. Histograms show the distribution of neutralization breadth for Abs with full seeding (upper row) and subset seeding (lower row) in (A) Scheme II—G|v1+v2, see 1 Ag (filled bars) and see both Ag (unfilled bars), and in (B) Scheme III—G|v1|v2. The height of a bar indicates the fraction of Abs having a particular breadth.

**Fig 4:**
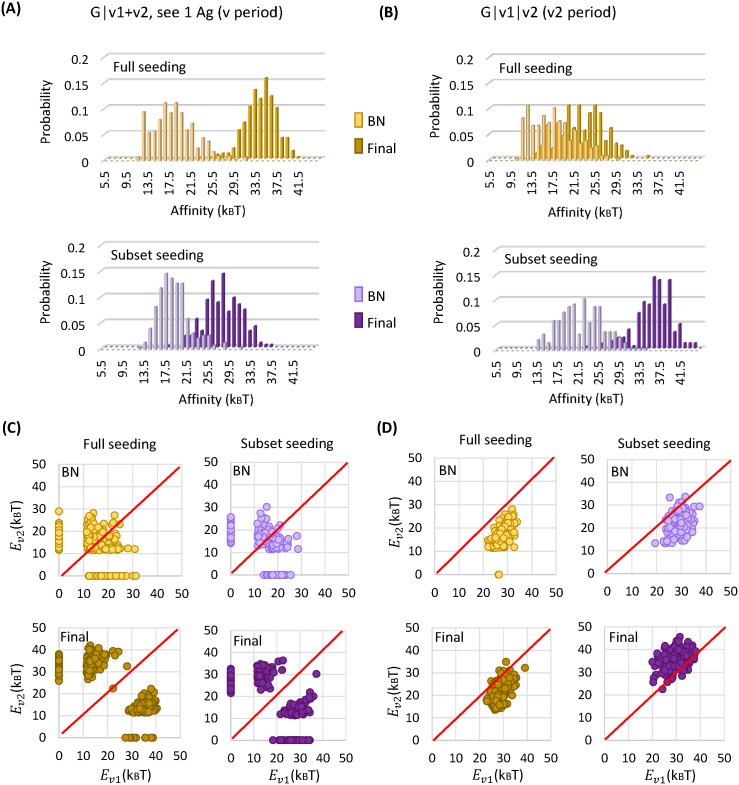
Effect of seeding diversity on maturation efficiency and antibody specificity. Results are shown for bnAb-producing schemes: G|v1+v2, see 1 Ag (left) and G|v1|v2 (right). (A-B) Distribution of B cell affinity for encountered Ag, at the population bottleneck (BN) and the end of maturation (Final), i.e., the start and the end of the post-bottleneck stage. Loss in seeding diversity slows adaptation in G|v1+v2, see 1 Ag, yet prolongs maturation in G|v1|v2. (C-D) Mean affinity of B cells that are reactive to one (symbols along either axis) or both (symbols away from both axes) of the variants, at the population bottleneck (BN) and the end of maturation (Final). Same conditions as in (A-B). Each symbol represents a surviving GC. Red diagonal lines indicate equal affinity to the variants, i.e., *E*_*v*1_ = *E*_*v*2_. Mature B cells in G|v1+v2, see 1 Ag are highly specific for one of the variants, whereas those in G|v1|v2 are reactive to both variants with comparable high affinities.

If each mature GC population is passed on to the next immunization period as is (full seeding), two schemes can elicit bnAbs with high probabilities–G|v1+v2, see 1 Ag and G|v1|v2 (filled bars in upper panels of Fig 3). Although Abs resulting from these two schemes exhibit similarly large breadth, they develop distinct patterns of interaction with the variable residues via disparate evolutionary paths (S1 Fig). In scheme G|v1+v2, see 1 Ag, due to the conflict between selection forces and uncertainty in Ag encounter, almost all the surviving GCs evolve highly specific B cells for one of the variants (Fig 4C lower panels, symbols along either axis and two blobs far from the diagonal on either side)—some are barely responsive to the unfavorable variant (see an example in S1A Fig bottom panel) while others are not reactive at all, similar to those developed in scheme G|v1+v2, see both Ag (S1B Fig bottom panel). Here large breadth comes from strong binding to common mutations in the test panel sequences. One caveat is that we use a binary representation of the panel sequences which might lead to overestimate of the breadth of the Abs that are specific for particular mutations (S1 Text; S2 Fig). Therefore, the actual Ab breadth achievable by scheme G|v1+v2, see 1 Ag is upper bounded by the values shown in Fig 3A. Another consequence of specific interaction with variable residues is loss of prior specificity during the course of maturation; as B cell clones specific for different Ag variants compete for dominance in a GC, this polyclonal population can partially lose its reactivity to earlier variants as new specificity develops (marked by asters in S1A Fig middle panel).

Whereas in scheme G|v1|v2, since conflicting selection forces are separated in time, maturing B cells progressively weaken interactions with one set of variable residues and then another, so they acquire recognition of new variants without losing responsiveness to old ones, i.e., cross-reactivity expands in range (S1C Fig middle and bottom panels). Resulting B cells bind to both variants with comparable affinities well-above the activation threshold (Fig 4D lower panels, a single blob close to the diagonal). Here moderate interactions with all the variable residues along with strong contact to the conserved core give rise to a large breadth.

Curiously, however, loss in initial diversity (subset seeding) has opposite effects on breadth development in these two bnAb-producing schemes (Fig 3). In scheme G|v1+v2, see 1 Ag, diversity loss broadens the distribution of breadth toward the lower end (Fig 3A, filled yellow bars to filled purple bars), whereas in scheme G|v1|v2, an even narrower distribution toward the largest breadth results (Fig 3B, yellow to purple bars).

**Fig 5:**
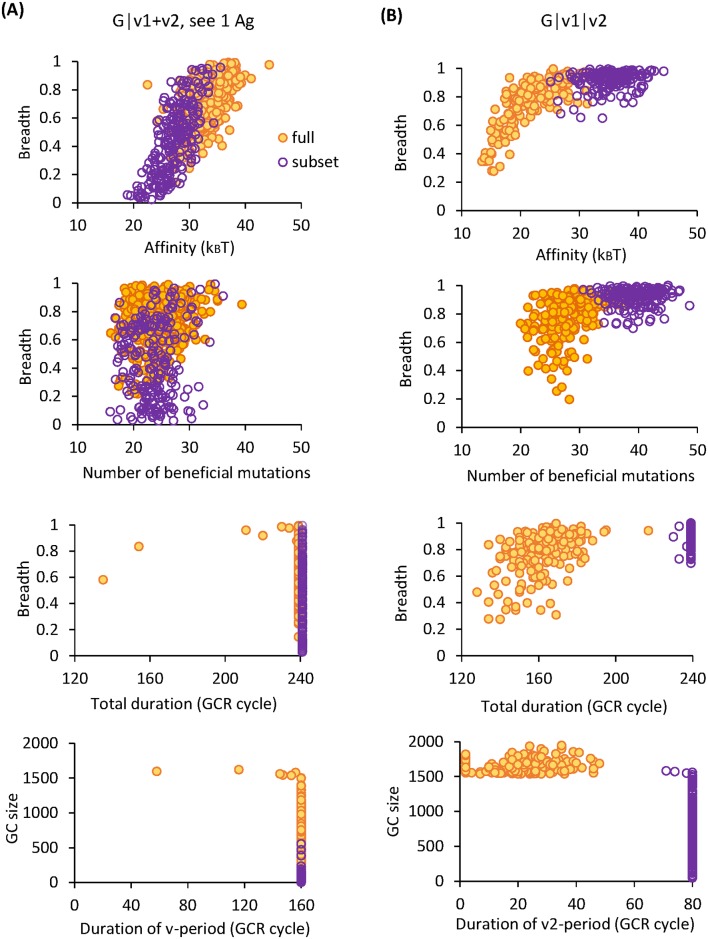
Potent bnAbs develop through persistent maturation at intermediate population sizes. Correlations between GC characteristics (size and duration) and the efficacy of bnAb induction (breadth and affinity of resulting Abs) are shown via scatter plots of the measures, contrasting full seeding (yellow filled symbols) and subset seeding (purple open symbols) for (A) G|v1+v2, see 1 Ag and (B) G|v1|v2. Each symbol represents a surviving GC. Top to bottom: Ab breadth versus affinity for encountered Ag, breadth versus number of beneficial mutations per surviving B cell, breadth versus total duration of GC reaction, and GC size versus duration of the v-period. Note in G|v1+v2, see 1 Ag, for both seeding schemes, almost all the surviving GCs reach the maximum duration (240 reaction cycles), yet subset seeding results in very small population sizes (too much frustration) and hence slow adaptation, leading to lower affinity and breadth. Whereas in G|v1|v2, while full seeding leads to brief maturation (too little frustration), subset seeding sustains the reaction and promotes concomitant development of high affinity and large breadth, both conferred by the large number of beneficial mutations.

Interestingly, these opposite trends originate from departing from versus approaching to persistent GC reactions. Effective affinity enhancement and breadth development both occur at intermediate population sizes, as in G|v1+v2, see 1 Ag with full seeding (Fig 5A filled symbols) and in G|v1|v2 with subset seeding (Fig 5B open symbols), where efficient and sustained maturation brings about increasingly more potent and broad mutants. To the contrary, too small GC sizes limit clonal diversity and strong genetic drift slows the incorporation of large-effect beneficial mutations into the population (G|v1+v2, see 1 Ag, subset seeding, Fig 5A open symbols), whereas too large GC sizes only allow brief maturation times before the Ag is consumed and the GC reaction wanes (G|v1|v2, full seeding, Fig 5B filled symbols), so neither situation is likely to evolve bnAbs. GC dynamics shows consistent trends (Fig 4): For G|v1+v2, see 1 Ag (Fig 4A), compared to full seeding (upper panel), subset seeding (lower panel) leads to slightly stronger mean affinity at the population bottleneck (BN), yet much weaker affinity when GCs mature (Final), due to slow adaptation at small population sizes. In contrast, in the v2-period of G|v1|v2 (Fig 4B), while for full seeding (upper panel) mean affinity only improves modestly when GC reactions end, subset seeding (lower panel) sustains maturation, resulting in significant affinity enhancement. Positive correlations between the efficacy of bnAb induction (affinity and breadth of resulting Abs) and the characteristics of surviving GCs (size and duration), as shown in Fig 5, make evident that persistent maturation plays a central role in evolving bnAbs, in harmony with the observation that potent bnAbs only arise after years of evolution driven by escaping viruses in chronically infected patients [16–20]. Rather than impede adaptation, diversity loss upon GC reseeding might provide a natural mechanism that encourages rare or de novo B cell clones and prolongs GC reactions in response to distant yet related variant Ags that arrive well apart in time.

Therefore, loss in seeding diversity renders scheme G|v1+v2, see 1 Ag even less efficacious due to too strong frustration, whereas reduced dominance of memory to past variants in the sequential scheme G|v1|v2 drives enduring adaptation and promotes acquisition of breadth-conferring mutations in a more effective manner.

### Effect of distracting epitopes on breadth development

How strongly a GC population is distracted from its target depends on how the selection forces represented by multiple variant Ags are temporally arranged. As we discuss in detail below, this interesting behavior stems from competition between the entropic advantage of D-targeting clones due to the constant presence and greater abundance of D epitopes, and the energetic advantage of T-targeting clones that accumulates through AM in a favorable environmental history. In the next section, we construct a simple analytical model to describe the competitive evolution of T- and D-targeting lineages, by translating the entropy-energy competition into trade-offs between average gain and variations in fitness. Here we focus on how the presence of distracting epitopes influences Ab breadth development using our individual-based simulations.

**Fig 6:**
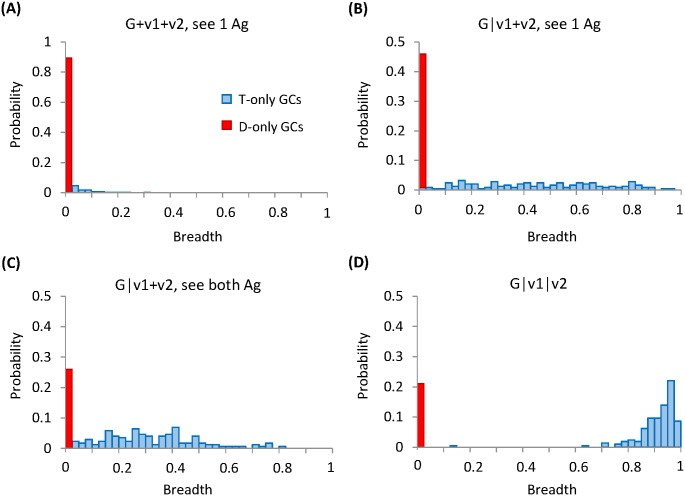
Effect of distracting epitopes on breadth development. Histograms show the distribution of antibody breadth for surviving GCs in various schemes. Height of the red bar at zero breadth indicates the fraction of surviving GCs that are dominated by D-targeting B cells, i.e.,%D-only GCs, which measures the degree of distraction. Whereas blue bars denote surviving GCs fully occupied by T-targeting B cells (i.e., T-only GCs). While scheme G+v1+v2, see 1 Ag (A) corresponds to the most frustrating scenario for T-targeting clones, exhibiting the lowest breadth and the highest chance of distraction, scheme G|v1|v2 (D), under optimally frustrating conditions, leads to the largest breadth and the least amount of distraction. Schemes G|v1+v2, see 1 Ag (B) and see both Ag (C) result in intermediate levels of breadth and distraction.

When 3 variants are presented in a cocktail (scheme I—G|v1+v2), T clones have no energetic advantage to start with, and suffer from a lower effective Ag concentration compared to D clones, since the T epitopes differ between variants. As a result, all the surviving T-targeting B cells are derived from GCs that are exclusively seeded by T clones. In these GCs, T lineages survive by alternately encountering G Ag and their favored variant among the two. Moderate interactions with the conserved residues and considerable footprint on the variable region of the target epitope leave the resulting Abs with small breadth (Fig 6A blue bars). In contrast, the majority of D lineages readily survive by binding to their selecting epitope present on both variants. Consequently, D clones win over T clones whenever they coexist in the seeding stage, resulting in dominance of D-targeting B cells in the mature pool (red bar at zero breadth in Fig 6A). Moreover, the overall GC survival rate dramatically increases compared to having only the T-epitopes, since D-targeting clones which win the competition and take over the population could save an otherwise deemed-to-collapse GC. This suggests that distraction might underlie the observation that Ab responses that emerge early in HIV infection are not broadly neutralizing [54].

If maturation first proceeds against G Ag (scheme II—G|v1+v2), T targeting clones would have acquired an energetic advantage by the time they face the variants, which alleviates their susceptibility to distraction (Fig 6B) as compared to in the cocktail scheme (Fig 6A). However, an initial advantage does not guarantee future success. Spatial heterogeneity in Ag distribution on the FDCs makes the number and type of encountered Ag uncertain for individual B cells in successive cycles of selection—each B cell can only see one type of Ag (see 1 Ag) if two variants tend to segregate, yet can encounter both (see both Ag) if two variants are well-mixed. The uncertainty in Ag encounter can be detrimental or even fatal to T-targeting lineages, if the fitness fluctuations due to v1-v2 alternation render T-targeting clones less fit than D-targeting clones sooner or later; as seen in S3A Fig, the worst possible mean affinity of T-targeting seeding clones often falls below that of D-targeting clones, which occurs when most T-targeting clones encounter their unfavorable variants. Surviving T-targeting lineages are the lucky ones that keep seeing their activating Ag in successive selection cycles, thus generating highly specific T clones. In addition, unfrustrated D lineages (i.e. having constant access to selecting epitopes) raise the risk of extinction for T lineages which, on their own, might have struggled through the bottleneck and produced neutralizing Abs upon maturation. This results in stronger susceptibility to distraction as well as lower affinity of T-targeting clones (S4A versus S4B Fig), if individual B cells encounter one type of Ag as compared to encountering both (a higher zero-breadth bar in Fig 6B than in Fig 6C). When both variants are accessible, all the B cells can see their selecting epitopes and optimize interactions iteratively. Most surviving GCs are dominated by T targeting B cells specific for mutated residues on their selecting epitopes, thus producing Abs with limited breadth (blue bars in Fig 6C).

When 3 variants are administered sequentially (scheme III—G|v1|v2), T-targeting clones also gain constant access to their activating variants in every encounter. In this case, as long as T and D clones coexist in the seeding population, D clones will be driven to extinction before reaching the bottleneck, thanks to sufficient fitness advantage of T clones accumulated in a serial fashion (panels C and D in S3 and S4 Figs). Upon immunization with v1, in most T-winning GCs, T clones only have modestly higher affinity than D clones to start with (S3C Fig). By the time v2 is administered, however, T lineages have acquired much enhanced energetic advantage via efficient maturation (S4C and S4D Fig upper panels), shown as a shoulder at appreciable mean affinity differences between T and D clones (red arrow in S4D Fig), which further suppresses distraction in subsequent competitive evolution (S4C and S4D Fig lower panels). This efficient maturation stems in part from non-local coupling effect; T-targeting clones that shrink their footprint on the variable region and simultaneously strengthen contact to the conserved core would achieve greater affinity gain thus outcompeting specific clones that suffer affinity penalties as they enhance binding to variable residues. Owing to a lack of conserved elements on the D epitopes, D-targeting clones do not benefit from the coupling effect either. As a result, the mature B cell repertoire is enriched with cross-reactive T-targeting clones (blue bars in Fig 6D) with significant enhancement in mean affinity and affinity variation (S4D Fig upper panel). A small fraction of surviving GCs are filled with zero-breadth antibodies (red bar in Fig 6D). These GCs are exclusively seeded by D clones, which exhibit limited affinity variation and moderate mean affinity when GCR ends (S4D Fig lower panel). Our simulations also showed that, if GCs start with a greater number of distinct seeding clones (e.g. of order 10 rather than a few), as has recently been suggested by imaging and sequencing studies of GC dynamics [30], distraction would be slightly weaker in all schemes. However, the relative degree of distraction between the schemes remains similar (S5 Fig).

In brief, temporal patterns of conflicting selection forces determine the level of frustration that T-targeting clones have to cope with in the absence of distraction, which in turn dictates how likely D-targeting clones can invade and win in various situations. We speculate that, in the presence of distraction, there should still be an optimum level of frustration [39] that corresponds to persistent and efficient maturation and hence a maximum likelihood of evolving bnAbs.

### Environmental histories of Ag choose evolutionary paths of Ab

In schemes G|v1+v2, see both Ag and G|v1|v2, all the competing lineages evolve in an identical antigenic environment that remains unchanged during each immunization period. In scheme G|v1+v2, see 1 Ag, however, each B cell lineage can experience a distinct environmental history, by randomly encountering one of the two variants in each mutation-selection cycle. Competition between T and D targeting lineages arises because, while G-matured T-targeting clones could have higher mean fitness than D clones to start with, they may also suffer from uncertainty in subsequent Ag encounter that leads to considerable fluctuations in fitness along the evolutionary trajectories.

To understand how environmental fluctuations influence the outcome of competitive dynamics between T and D targeting lineages, we consider a simple evolutionary model of two competing species in a rapidly fluctuating environment, similar to Refs [60–63]. This would allow us to identify the dominant mode of Ab response that emerges from the competition and verify the behavior in various regimes with our individual-based simulations. Population dynamics of the two species is described by the following stochastic differential equations:

$$\begin{array}{c} \dot{N_{T}}=\left(r_{T}-\frac{N}{K}\right)N_{T}+\sqrt{\left(r_{T}+\frac{N}{K}\right)N_{T}}\;\xi_{T}\left(t\right)+N_{T}\sigma_{T}\eta_{T}\left(t\right),\\ \dot{N_{D}}=\left(r_{D}-\frac{N}{K}\right)N_{D}+\sqrt{\left(r_{D}+\frac{N}{K}\right)N_{D}}\;\xi_{D}\left(t\right)+N_{D}\sigma_{D}\eta_{D}\left(t\right). \end{array}$$ (4)

Environmental variability is incorporated by a time modulation of the birth rates, *r*_*T*_ and *r*_*D*_, of T and D clones, modelled as Gaussian white noises of strength *σ*_*S*_, i.e., *r*_*S*_(*t*) = *r*_*S*_ + *σ*_*S*_ *η*_*S*_(*t*), where $\langle \eta_{S}(t)\eta_{S}(t\prime )\rangle =\delta (t-t\prime )$, with *S* = *T*, *D* being the species label. Death rate is assumed identical for both species, increasing with total population size *N* = *N*_*T*_ + *N*_*D*_. The logistic growth of the population is bounded by its steady state size, *N*^⋆^ = *K*[*r*_*T*_ *x* + *r*_*D*_(1 − *x*)], where *x* = *N*_*T*_/*N* is the proportion of T clones. This saturation may account for density dependent ecological factors, such as exhaustible Ag on FDCs and limited T cell help, in the context of GC reaction. In addition to the environmental noise, demographic fluctuations originated from the discreteness of the evolving entities yield the term $\sqrt{\left(r_{S}+N/K\right)N_{S}}\xi_{S}\left(t\right)$, where 〈*ξ*_*S*_(*t*)*ξ*_*S*_(*t*′)〉 = *δ*(*t* − *t*′), with the variance being the sum of deterministic birth and death rates.

We obtain the 2D Fokker-Planck equation equivalent to the Langevin description (Eq 4) and marginalize it with respect to the total population size, by assuming that selection acts on a much longer time scale than that of population growth. This leads us to a 1D Fokker-Planck equation for *P*(*x*, *t*) (derivation in S1 Text) that describes the evolution of population composition in the presence of environmental and demographic fluctuations:

$$\begin{array}{ccc} \frac{\partial }{\partial t}P\left(x,t\right)= & - & \partial_{x}\left\{\left[s-\sigma_{T}^{2}x+\sigma_{D}^{2}\left(1-x\right)-\epsilon \sigma_{T}\sigma_{D}\left(1-2x\right)\right]x\left(1-x\right)P\left(x,t\right)\right\}\\ & + & \partial_{x}^{2}\left\{\left[\frac{\sigma_{T}^{2}+\sigma_{D}^{2}-2\epsilon \sigma_{T}\sigma_{D}}{2}x\left(1-x\right)+\frac{1}{K}\right]x\left(1-x\right)P\left(x,t\right)\right\}. \end{array}$$ (5)

Here *s* ≡ *r*_*T*_ − *r*_*D*_ denotes selection strength and the coefficient $\epsilon =\langle \eta_{T}\eta_{D}\rangle /\sqrt{\langle \eta_{T}^{2}\rangle \langle \eta_{D}^{2}\rangle }$ characterizes the correlation of environmental noises experienced by the two competing species. Note that even though T and D clones have independent histories of Ag encounter, i.e., environmental noises of the two species are uncorrelated (*ϵ* = 0), drift $v\left(x\right)=s-\sigma_{T}^{2}x+\sigma_{D}^{2}\left(1-x\right)$ and diffusion $D\left(x\right)=\left(\sigma_{T}^{2}+\sigma_{D}^{2}\right)x\left(1-x\right)/2+1/K$ both depend on environmental variability of T and D clones (*σ*_*T*_ and *σ*_*D*_) and population composition (*x*).

**Fig 7:**
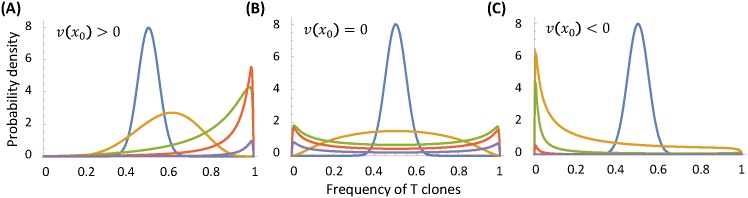
Susceptibility to distraction under fitness fluctuations. Distributions of the frequency of T clones are obtained from numerical integration of the 2-species model (Eq 5) with demographic noise and uncorrelated fitness noise (*ϵ* = 0). Distributions start from a Gaussian with mean *x*_0_ = 0.5 and width 0.05. Absorbing boundaries are applied and results shown at different times, t = 0 (blue), 1 (orange), 5 (green), 10 (red), and 20 (purple). A to C: *σ*_*T*_ = 0.5, 1, 2, corresponding to *v*(*x*_0_)>0, = 0, <0, respectively. Stronger fitness fluctuations increase the likelihood of extinction for T clones and accelerate relaxation. Coexistence (B) requires an exact balance of fitness bias and variations. Other parameters include *s* = 0.5, *σ*_*D*_ = 0 and *K* = 100.

Scenarios of interest in scheme G|v1+v2, see 1 Ag correspond to *s* = *r*_*T*_ − *r*_*D*_ ≠ 0 and *σ*_*T*_ > *σ*_*D*_ = 0. Fig 7 shows the distribution of T-clone abundance at different times (blue to purple: early to late) obtained from numerical integration of Eq (5). It starts from a Gaussian distribution centered at *x*_0_ = 0.5, i.e., similar proportion of two subpopulations. Absorbing boundary conditions are applied, so that vanishing to the right (left) boundary corresponds to fixation (extinction) of T clones. As shown, an increasing amplitude of environmental fluctuations (increasing *σ*_*T*_ from A to C) elevates the risk of extinction for T clones and speeds up relaxation. The condition $s-\sigma_{T}^{2}x_{0}=0$ (panel B) represents the boundary between the regimes where either T or D clones fixate; balanced bias and fluctuations result in strong uncertainty in clonal composition and most likely coexistence.

**Fig 8:**
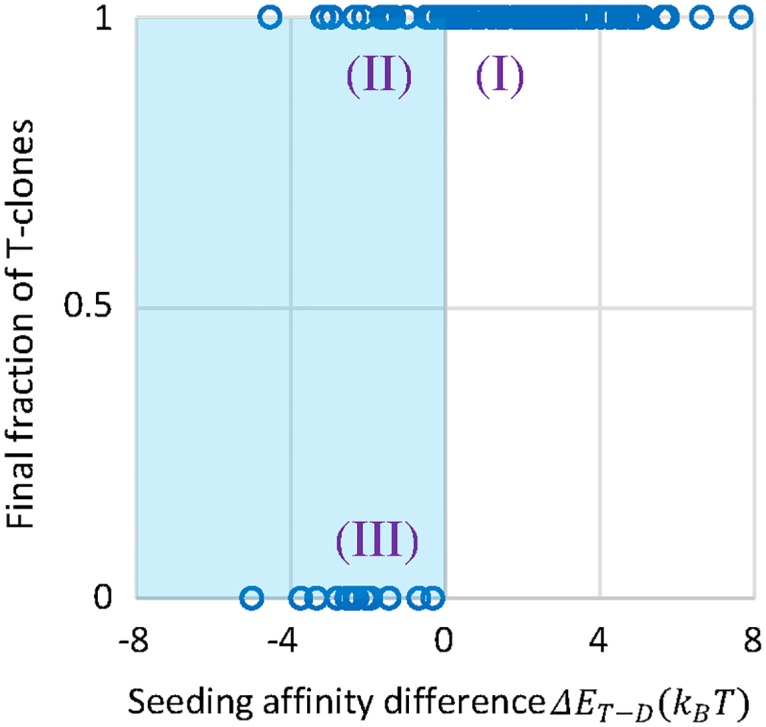
Clone fate in fluctuating environments. The scatter plot shows the dependence of clone fate, measured by the final percentage of T clones in a surviving GC, on the affinity difference between T and D seeding clones at the start of the v-period in scheme G|v1+v2, see 1 Ag. The affinity of a T clone is an average of affinities with respect to the two variants. Each symbol is a surviving GC. All the surviving GCs co-seeded by T and D clones are dominated by one subpopulation when mature, i.e., the final percentage of T clones is either 0% or 100%. Three regimes of clone fate are noted. Regime I (60% of surviving GCs): higher mean affinity of T seeding clones guarantees their dominance upon maturation. Regimes II and III (shaded area): lower mean affinity of T seeding clones leads to uncertainty in the dominant species. T-winning (regime II) and D-winning (regime III) cases occur with similar probabilities, 17% and 23%, respectively.

**Fig 9:**
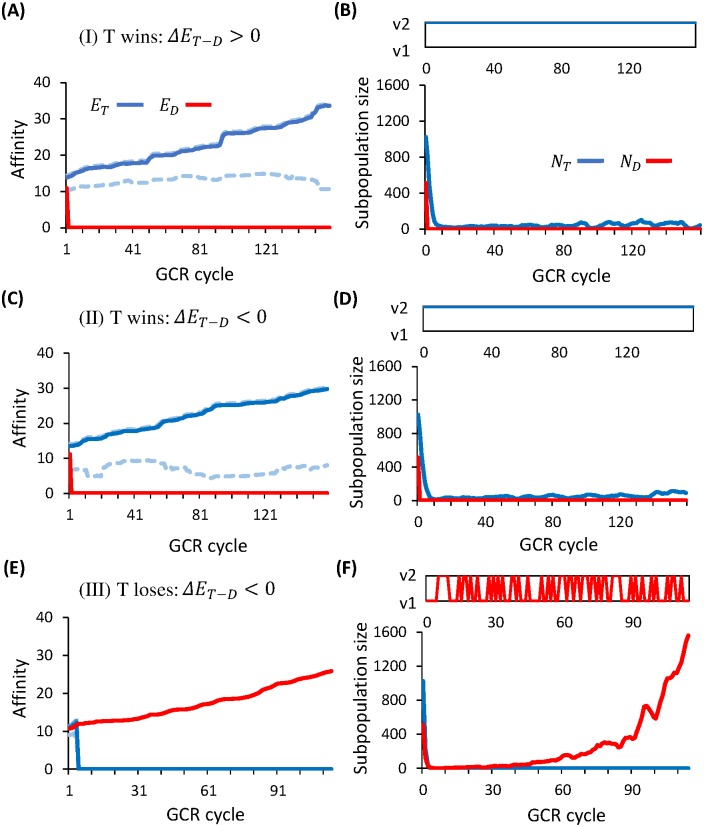
Competitive dynamics of T-targeting and D-targeting lineages. Representative evolutionary trajectories of T (red curves) and D (blue curves) subpopulations are shown for the three regimes noted in Fig 8, one GC for each regime. Time evolution of mean affinity (A, C, E) and size (lower panel of B, D, F) of two subpopulations are presented, along with a history of encountered Ag for a typical B cell lineage in the winning subpopulation (upper panel of B, D, F). The actual affinity of T clones (solid blue curve in A, C, E) is bounded by the best and worst possible affinities (dashed blue curves in A, C, E).

Possible clonal fate observed in our individual-based in silico model can be identified with the three regimes marked in Fig 8:

Regime I (60% of surviving GCs, corresponding to $s-\sigma_{T}^{2}x_{0}>0$): T clones win with a higher *mean* affinity (see example in Fig 9A and 9B) than D clones (*s* > 0) and vanishing fluctuations (*σ*_*T*_ = 0). In this regime, seeding T clones have a higher mean affinity than D clones (Fig 9A), and they rapidly take over the population in approach to the population bottleneck. This is achieved by persistent encounters with the favorable variant (upper panel of Fig 9B), thereby closely tracing the best possible affinity (solid blue curve in Fig 9A), in a subset of T-targeting lineages. Yet the GC size remains very small (lower panel of Fig 9B) since T-targeting lineages that experience fluctuating environments are outcompeted and absent from the recycled population—even if T clones win. Thus, resulting Ab titers are low.

Regime II (17% of surviving GCs, corresponding to $s-\sigma_{T}^{2}x_{0}>0$): T clones win with a higher *best* affinity (see example in Fig 9C and 9D) than D clones (*s* > 0) and steady environments (*σ*_*T*_ = 0). In this regime, seeding T clones have slightly lower mean affinity than D clones (Fig 9C), and in particular, the worst possible affinity (dashed line) is constantly below the activation threshold. Nonetheless, as long as a subset of T lineages keep seeing their favorable variant, D lineages are quickly driven to extinction. Again the GC size stays small (lower panel of Fig 9D), since in each GCR cycle a considerable fraction of T-targeting lineages encounter the non-activating variant and apoptose.

Regime III (23% of surviving GCs, corresponding to $s-\sigma_{T}^{2}x_{0}<0$): T clones lose due to strong environmental variabilities in spite of a modest selective advantage for persistent best affinity. Here the environmental fluctuations lead to a sudden catastrophe at early times (blue curve in Fig 9E), reducing the reproduction rate of T-targeting clones to a value which can no longer sustain a steady (sub)population (blue curve in Fig 9F). In this regime, seeding D clones have slightly higher mean affinity, and even the best possible affinity of T clones (solid blue curve in Fig 9E) is barely above the activation threshold. Therefore, once most T-targeting lineages encounter their non-activating variants, D clones take over the population. Since alternate encounter of v1 and v2 (upper panel of Fig 9F) incurs no affinity cost to the D subpopulation, it grows to the GC capacity (red curve in the lower panel of Fig 9F).

T-D coexistence is not observed in any mature GC from our simulations so far, because the simulated system is located in a strong-selection regime with a deep population bottleneck: Strong selection discerns small affinity differences between B cell clones, whether they target the same or different epitopes, leading to the dominance of a single subpopulation that targets a particular epitope. Nonetheless, inter-GC variations provide an overall diversity of Ag specificities in the mature B cell repertoire. In reality, however, T- and D-targeting clones may well coexist in maturing GCs. Indeed, as we weaken selection by increasing Ag concentrations as well as decreasing the number of neighboring B cells competing for T cell help, simultaneous maturation of T and D lineages appear in all schemes (S6 Fig). This is because higher Ag concentrations effectively lower the activation threshold for B cells and also trigger a stronger response of helper T cells, whereas a smaller number of competitors for each B cell in getting T cell help makes it easier for mediocre B cells to survive, as they manage to survive selection by not competing with the strongest binders. Note that each subpopulation targeting a particular epitope contains multiple clones (S6 Fig bottom row), and that specificity loss mostly occurs during the population bottleneck (S6A and S6C Fig middle panels).

### Optimal Ag dosage nurtures cross-reactivity and suppresses distraction

The sequential scheme is not unconditionally superior—its efficacy relies on sustained maturation against each variant, which only occurs within a window of Ag concentration, where GC survival and Ab breadth both peak and coincide [39]. For a given number of Ag variants with properly chosen mutational distances separating them, Ag dose controls the size and duration of GCs and hence the efficiency of B cell adaptation. At an optimal Ag concentration, maturation takes place in a slowly yet steadily expanding population following the population bottleneck (S1C Fig upper panel): a modest rate of Ag consumption allows enough time for beneficial mutations to enter the population and in the meanwhile, an intermediate population size promotes efficient selective spread of beneficial mutations without causing much clonal interference. As a result, cross-reactive clones that have accumulated the large number of beneficial mutations emerge before Ag is exhausted and GCs disassemble (arrows in S1C Fig). In this manner persistent reaction and efficient adaptation can be achieved simultaneously.

To make these arguments more concrete, we relate population dynamics of a GC to the expected selection probability as follows. If each B cell divides *r* times per cycle with a probability *p*_*L*_ to acquire lethal mutations, then the population- and time-averaged selection probability ${\bar{P}}_{sel}$ can be estimated by ${\left[2^{r}\times \left(1-p_{L}\right)\times {\bar{P}}_{sel}\right]}^{t_{f}-t_{BN}}=N_{f}/N_{BN}$, where *t*_*f*_(*t*_*BN*_) and *N*_*f*_(*N*_*BN*_) denote the time and GC size at the end (population bottleneck) of a maturation period, respectively. As described above, at an optimal Ag dose, B cell populations undergo sustained adaptation following the bottleneck and reach GC capacity *N*_*max*_ at the maximum GCR duration *t*_*max*_, i.e., *N*_*f*_ = *N*_*max*_ at *t*_*f*_ = *t*_*max*_. This gives the expression of the corresponding optimal selection probability $P_{sel}^{⋆}={\left[\frac{N_{max}}{N_{BN}}\right]}^{\frac{1}{t_{max}-t_{BN}}}/[2^{r}\times \left(1-p_{L}\right)]$. If *r* = 2, *p*_*L*_ = 0.3, and a 100-fold increase in population size occurs during 70 GCR cycles, we would have $P_{sel}^{⋆}≃0.38$. This value is very close to that computed from our stochastic simulations of the sequential scheme, based on the selection probability of individual B cells in each cycle $P_{sel}^{i}\left(t\right)=P_{a}^{i}\left(t\right)\cdot P_{Th}^{i}\left(t\right)$, where $P_{a}^{i}$ and $P_{Th}^{i}$ are given by Eqs (1) and (2). This matching indicates that Ag concentrations used in our in silico study indeed lie within the optimal range leading to persistent maturation. Therefore, an appropriate range of Ag dosage would invoke an optimal strength of selection.

In addition to facilitating breadth development, an optimal Ag dose also serves to suppress distraction. In synergy with the sequential procedure, it helps to enhance the selective advantage of increasingly more focused T clones, thus driving D lineages to extinction prior to the population bottleneck. In contrast, if Ag concentration is higher than optimal, as seen in S6 Fig, D-targeting lineages are more likely to survive the bottleneck and coexist with T lineages that have acquired little fitness advantage during brief AM in earlier periods. Therefore, GC populations are more susceptible to distraction even when environments do not fluctuate, and T lineages are more prone to extinction as a new Ag variant arrives.

## Discussion

In facing highly mutable complex pathogens, such as HIV, affinity maturation of Abs is not only frustrated by conflicting selection forces, but also distracted by immunodominant variable epitopes. Design of immunogens and efficient immunization protocols to elicit bnAbs that target the conserved part of the pathogen requires a detailed understanding of the selection forces that arise from multiple Ag variants each carrying multiple epitopes and follow various temporal procedures. To this end, we develop a stochastic dynamic model of affinity maturation driven by distinct yet related Ag variants with complex epitopes, identify the evolutionary conditions that favor the selection of potent bnAbs and deduce immunization strategies that can potentially resolve conflict and minimize distraction at the same time.

Protecting against highly mutable pathogens is likely to require multiple immunizations with distinct immunogens. Two new aspects become important to understand—loss of B cell diversity in between periods of immunization, and the presence of distracting epitopes that might lead to seeding or invasion of GCs by D-targeting B cells. Surprisingly, our in silico results suggest that loss in B cell diversity, upon sampling from the memory pool to seed nascent GCs, does not necessarily impede adaptation; instead, it can facilitate a broader search of the sequence space and prolong the consumption of FDC-Ags, which allows persistent evolution of favorable mutations that confer breadth. An underlying broad distribution of intermediate GC sizes is shown to lead to large breadth and great diversity of the mature B cell repertoire, benefiting from efficient adaptation (if too small GCs, slow adaptation) and sustained reaction (if too large GCs, brief maturation). Although Ag is not self-renewing in immunization settings, appropriate temporal arrangements of selection forces can serve to maintain effective adaptation.

We find that, in the presence of distracting epitopes, how selection forces are arranged in time determines the relative selective advantage between T and D lineages that compete for survival, and thus the susceptibility of a GC population to distraction. D-targeting clones have the best chance of invasion in the cocktail scheme, where T-targeting clones are severely frustrated by mutationally distant T epitopes present on the three Ag variants, and the dominance of D-targeting clones leads to very narrow (autologous) Ab response. If one primes with G Ag and then boosts with a mixture of two variants, highly strain-specific Abs result from T lineages that have constant access to their selecting epitopes. But Ab titers are not high, because in each cycle of mutation and selection, a large fraction of T clones encounter the non-activating Ag and go extinct. Only when Ag variants are administered sequentially, bnAbs are likely to evolve efficiently. On the one hand, by separating conflicting selection forces in time, it favors cross-reactive lineages over specific ones. On the other hand, by aiding in serial enhancement of selective advantage for increasingly more focused T clones, it thwarts distracted lineages. Therefore, the same set of beneficial mutations that confer breadth to T clones also grant them affinity superiority over D clones, which suggests sequential immunization with an optimal design should make possible simultaneous achievement of large breadth and little distraction. Experimental measurement of the relative abundance and affinity of T-epitope and D-epitope directed Abs in the serum following different immunization schemes may be able to test the predictions that we report in this paper.

An optimal range of Ag dose is important for the effectiveness of the sequential scheme. For a given set of variant Ags, an optimal dose enhances GC survival while preventing cross-reactive memories from rapidly occupying nascent GCs. Even if the range of optimal Ag concentration is relatively narrow, since Ag distribution is heterogeneous within and between GCs, an appreciable fraction of B cells are likely to experience an optimal dose. In other words, spatial heterogeneity of Ag concentration could effectively broaden the range of optimality. Ag dose also influences the degree of frustration when multiple variants are present. In this study, we give each B cell one shot to bind and internalize Ag. If multiple shots are allowed and the variety of Ag is small, frustration will not arise. This resembles the “See all Ag” case, which should usually result in strain-specific Abs [39]. However, reducing Ag concentration while allowing multiple shots will frustrate the system again in the “See 1 Ag” case.

In contrast to past works which assume that conserved and variable elements constitute separate epitopes that occupy different locations on the Env protein, we model the target epitope (e.g. CD4 binding site of HIV-1) as containing simultaneously conserved and variable residues in physical proximity, such that low-affinity precusor B cells must evolve to evade the variable occluding elements in order to access the conserved core. Reducing interactions with variable residues compensated by enhancing contact to the conserved elements is critical for breadth development. However, these correlated changes of interactions take many random mutational trials to occur and fixate. Very often, potentially broad lineages are purged by distracted or specific ones early in response. In order to grant bnAb precusors immediate advantage over distracted ones, we may create an environment where T-clones can emerge and establish in the first place. This can be realized by priming with solely G Ag, an engineered scaffold of the Env trimer that only presents the target epitope in its non-mutated form and activates the desirable germline T clones. To further favor broad T clones over specific ones, a sequential presentation of Ag variants with shared conserved part yet distant variable part of the T epitope would selectively expand cross-reactive T lineages while filtering out specific clones. An appropriate choice of Ag dose and antigenic distances between the variants would prolong adaptation of T lineages with minimal extinction; energetic advantage of T clones thus accumulates across successive maturation periods, beating the entropic disadvantage. In sum, in the sequential scheme, desirable germline T clones get activated and undergo sustained maturation against G Ag to develop cross-reactivity to v1 and outcompete naïve D clones, followed by effective adaptation to respond to v2, thereby winning over naïve and v1-matured D clones, as well as v1-specific T clones.

This flexible in silico model offers ample room for optimization of the immunization strategies in future studies. One immediate direction is a potential combination strategy that integrates sequential and mixture schemes: after activating the appropriate germline B cells, if we are allowed multiple doses of different mixtures, we can imagine gradually increasing the mutational distance between the variants in consecutive mixtures, which likely mitigate frustration at early stages of affinity maturation and enable a smoother broadening of cross-reactivity with even better success rates, compared to well-separated variants one at a time. A new report [44] on sequential immunization with distant variants followed by a mixture of closer variants suggests an alternative approach. To better guide the choice of immunogens and to identify the optimal conditions for a combination strategy, further computational investigations are needed to complement experimental studies.

Practically, the results of this paper should have useful implications for immunization against other highly mutable pathogens (e.g. malaria) that might require multiple vaccinations. Conceptually, this work poses interesting theoretical questions in evolutionary dynamics for future exploration, namely, how to focus evolutionary paths under the constraints of diversity of selection forces and distracting selection pressures.

## Supporting Information

S1 Text Supplemental information and detailed analytical derivations. (A) Polymorphism of variable residues in the test panel sequences. (B) Fokker-Planck equation describing competitive evolution of T and D targeting lineages.
(PDF)

S1 Fig Development of cross-reactivity in bnAb-producing schemes with full seeding. Shown are temporal trajectories of cross-reactivity and affinity for representative GCs in schemes G|v1+v2, see 1 Ag (A), G|v1+v2, see both Ag (B) and G|v1|v2 (C). Each column is one GC. Top to bottom panels display respectively the number, fraction and mean affinity of reactive B cells to the three variants, G (blue), v1 (orange) and v2 (grey). Blue arrows mark the introduction of both variants simultaneously (A and B) or one variant at a time (C). Red stars in (A) indicate temporary partial loss in reactivity of a maturing population to one of the variants, due to competing lineages with different specificities. Red arrows in (C) point to the emergence of mutants that are reactive to the soon-to-arrive variant. Note that as reactivity to a new variant develops (e.g. when blue/orange gives way to gray), responsiveness to older ones is retained.
(EPS)

S2 Fig Site entropy of variable residues in the test panel sequences. The entropy of site *i* is defined as *S*_*i*_ = −∑_*a*_ *f*_*i*,*a*_ log_2_ *f*_*i*,*a*_, where *f*_*i*,*a*_ denotes the frequency of amino acid a at site *i*. The entropy of a binary site is no more than 1 (e.g. red symbols). Thus a large site entropy indicates significant polymorphism, i.e., there are appreciable contributions from more than 2 amino-acid letters in that position (purple symbols).
(EPS)

S3 Fig Affinity difference between T and D seeding clones. Histograms show the distribution of affinity difference between T and D seeding clones for surviving GCs in (A) G|v1+v2, see 1 Ag, (B) G|v1+v2, see both Ag, G|v1|v2, v1-period (C) and v2-period (D). Affinity values are averaged within T or D subpopulation. In (A), blue (grey) corresponds to the best (worst) affinity of a T subpopulation, where all the T seeding clones encounter their favorable (unfavorable) variants. The red arrow in (D) points to a shoulder that emerges at large affinity difference at the beginning of the v2-period in scheme G|v1|v2, indicating accumulation of selective advantage in T-targeting lineages during maturation against v1.
(EPS)

S4 Fig Affinity maturation of T and D targeting subpopulations. Histograms of mean affinity of T-targeting (*E*_*T*_, upper panels) and D-targeting (*E*_*D*_, lower panels) B cell clones in surviving GCs at the beginning (*E*^*i*^, blue), bottleneck (*E*^*BN*^, orange) and end (*E*^*f*^, grey) of maturation are shown for G|v1+v2, see 1 Ag (A) and see both Ag (B) and for G|v1|v2, v1-period (C) and v2-period (D). Note that maturation against G has been completed. Height of the bars in the leftmost bin corresponds to the fraction of all-D (upper panels) and all-T (lower panels) GCs among the surviving ones. At the initial time (blue bars), the total probability of all-T and all-D GCs (leftmost bins) is smaller than 1, since the rest are co-seeded by T and D clones. In G|v1+v2, see 1 Ag (A), a higher proportion of all-D seeded GCs (a taller leftmost blue bar in the upper panel) compared to other schemes reflects a higher survival rate of all-D seeded GCs than all-T and T-D seeded ones in this scheme. T and D clones have similar affinity distribution upon maturation in scheme G|v1+v2, see 1 Ag (A), whereas in other schemes (B-D), mature T clones have higher mean affinity and greater affinity variation than mature D clones.
(EPS)

S5 Fig Effect of a greater number of seeding clones. Histograms show the distribution of antibody breadth for (A) G|v1+v2, see 1 Ag and (B) G|v1|v2, both seeded with *N*_*sd*_ = 12 distinct clones. In both schemes, distraction is weaker (red bars being shorter) compared to seeding with *N*_*sd*_ = 3 clones (panels B and D of Fig 6 in Main Text). However, the relative degree of distraction between the schemes remains similar.
(EPS)

S6 Fig Coexistence of T and D targeting lineages under weaker selection pressures. Temporal trajectories of simultaneously maturing T and D lineages are shown for G+v1+v2, see all Ag (A), v-period of G|v1+v2, see both Ag (B) and v1-period of G|v1|v2 (C). Each column is a typical GC. Top to bottom panels respectively show the size of subpopulations targeting each of the available epitopes (color coded), relative abundance of selecting epitopes (same color scheme as the top row) and the frequencies of top five B cell clones (lighter to darker hue for larger to smaller clones). Note that a subpopulation targeting a particular epitope often contains multiple clones. Post-bottleneck coexistence of T and D targeting lineages is made possible by increasing Ag concentrations and reducing the number of competitors for each B cell in receiving T cell help.
(EPS)
