# Supplementary material for: Optimal Sequential Immunization Can Focus Antibody Responses against Diversity Loss and Distraction
Source: PLoS Comput Biol. 2017 Jan 30;13(1):e1005336. doi: 10.1371/journal.pcbi.1005336 (PMC5279722; doi:10.1371/journal.pcbi.1005336)
Supplement: S1 Text — (A) Polymorphism of variable residues in the test panel sequences. (B) Fokker-Planck equation describing competitive evolution of T and D targeting lineages. (PDF) [file pcbi.1005336.s001.pdf]

S1 Text: Optimal Sequential Immunization Can Focus Antibody Responses against Diversity Loss and Distraction

Shenshen Wang

A. Polymorphism of variable residues in the test panel sequences

We evaluate Ab breadth using a binary representation of the panel sequences, i.e., any non-WT amino acids are treated equally as mutated ($s_k = -1$). This might overestimate the breadth of the Abs that are specific for particular mutations, if the corresponding sites happen to have strong polymorphism. We quantify this potential deviation with site entropy of the variable residues in the panel sequences, defined as $S_i = -\sum_a f_{i,a} \log_2 f_{i,a}$, where $f_{i,a}$ is the frequency of amino acid a at site i. The entropy of a binary site is no more than 1. As seen in S2 Fig, most variable residues exhibit a large site entropy indicating significant polymorphism, i.e., there are appreciable contributions from more than 2 amino acid letters in those positions. Therefore, the actual Ab breadth that can be achieved by scheme G|v1+v2, see 1 Ag is upper bounded by the values shown in Fig 3A (Main text).

B. Fokker-Planck equation describing competitive evolution of T and D targeting lineages

The Fokker-Planck equation equivalent to the Langevin description (Eq 4 in Main Text) is given by

$$\begin{aligned}\frac{\partial}{\partial t} P(N_T, N_D, t) =& -\sum_{S=T,D} \partial_{N_S} \left[\left(r_S - \frac{N}{K} \right) N_S P \right] \\ &+ \frac{1}{2} \sum_{S=T,D} \partial^2_{N_S} \left\{ \left[(N_S \sigma_S)^2 + N_S \left(r_S + \frac{N}{K} \right) \right] P \right\} \\ &+ \epsilon \partial_{N_T} \partial_{N_D} \sigma_T \sigma_D N_T N_D P. \end{aligned} \tag{1}$$

Here the drift term describes the deterministic logistic growth, whereas the diffusion terms sum up the two types of fluctuations. There is also a contribution from the correlation of environmental noises experienced by the two species, with a correlation coefficient $\epsilon = \langle \eta_T \eta_D \rangle / \sqrt{\langle \eta_T^2 \rangle \langle \eta_D^2 \rangle}$ between -1 and 1.

Since we are interested in the evolution of population composition in the presence of environmental and demographic noises, it is more convenient to switch to the continuous variable $x = N_T/N$ and obtain the equivalent 2D Fokker-Planck equation for $P(N, x, t)$:

$$\begin{aligned}\frac{\partial}{\partial t} P(N, x, t) =& \epsilon \left[\frac{x(1-x)}{N} \partial_x^2 + N \partial_N^2 + (1-2x) \partial_x \partial_N \right] N x (1-x) \sigma_T \sigma_D P(N, x, t) \\ &- \left[(1-x) \partial_x + N \partial_N \right] \left(r_T - \frac{N}{K} \right) x P(N, x, t) \\ &+ \left(x \partial_x - N \partial_N \right) \left(r_D - \frac{N}{K} \right) (1-x) P(N, x, t) \\ &+ \frac{1}{2} \left[\frac{(1-x)^2}{N} \partial_x^2 + N \partial_N^2 + 2(1-x) \partial_x \partial_N \right] \left[N x^2 \sigma_T^2 + \left(r_T + \frac{N}{K} \right) x \right] P(N, x, t) \\ &+ \frac{1}{2} \left[\frac{x^2}{N} \partial_x^2 + N \partial_N^2 - 2x \partial_x \partial_N \right] \left[N (1-x)^2 \sigma_D^2 + \left(r_D + \frac{N}{K} \right) (1-x) \right] P(N, x, t). \end{aligned} \tag{2}$$

By assuming that selection acts on a much longer time scale than that of population growth, i.e. $s \equiv r_T - r_D \ll r_T x + r_D (1-x)$, we can marginalize Eq (2) with respect to N and arrive at a 1D Fokker-Planck equation for $P(x, t)$:

$$\begin{aligned}\frac{\partial}{\partial t} P(x, t) =& -\partial_x \left\{ \left[s - \sigma_T^2 x + \sigma_D^2 (1-x) - \epsilon \sigma_T \sigma_D (1-2x) \right] x (1-x) P(x, t) \right\} \\ &+ \partial_x^2 \left\{ \left[\frac{\sigma_T^2 + \sigma_D^2 - 2\epsilon \sigma_T \sigma_D}{2} x (1-x) + \frac{1}{K} \right] x (1-x) P(x, t) \right\}. \end{aligned} \tag{3}$$
